# Economic and Social Standing of Individuals in Iran Diagnosed with Multiple Sclerosis

**DOI:** 10.34172/aim.2023.63

**Published:** 2023-08-01

**Authors:** Fereshteh Ghadiri, Mohammad Ali Sahraian, Fereshteh Ashtari, Seyed Mohammad Baghbanian, Nastaran Majdi-Nasab, Hamidreza Hatamian, Fardin Faraji, Asghar Bayati, Ehsan Sharifipour, Nazanin Jalali, Hossein Mozhdehipanah, Hoda Kamali, Saeideh Ayoubi, Sharareh Eskandarieh, Abdorreza Naser Moghadasi

**Affiliations:** ^1^Multiple Sclerosis Research Center, Neuroscience Institute, Tehran University of Medical Sciences, Tehran, Iran; ^2^Isfahan Neurosciences Research Center, Isfahan University of Medical Sciences, Isfahan, Iran; ^3^Department of Neurology, Booalicina Hospital, Mazandaran University of Medical Sciences, Sari, Iran; ^4^Musculoskeletal Rehabilitation Research Center, Ahvaz Jundishapur University of Medical Sciences, Ahvaz, Iran; ^5^Department of Neurology, School of Medicine, Poursina Hospital, Guilan University of Medical Sciences, Rasht, Iran; ^6^Department of Neurology, School of Medicine, Arak University of Medical Sciences, Arak, Iran; ^7^Department of Neurology, Shahrekord University of Medical Sciences and Health Services, Shahrekord, Iran; ^8^Department of Neurology, Shohada Tajrish Hospital, Shahid Beheshti University of Medical Sciences, Tehran, Iran; ^9^Department of Neurology, School of Medicine, Rafsanjan University of Medical Sciences, Rafsanjan, Iran; ^10^Department of Neurology, Qazvin University of Medical Sciences, Qazvin, Iran; ^11^Neurology Research Center, Kerman University of Medical Sciences, Kerman, Iran

**Keywords:** Iran, Multiple sclerosis, Socioeconomic status

## Abstract

**Background:**

Multiple sclerosis (MS) may be affected by socioeconomic status (SES). This study aims to explore the determinants of SES among Iranian patients with MS and examine how these factors relate to disability and disease progression.

**Methods:**

All patients with MS listed in the nationwide MS registry of Iran (NMSRI) until January 8, 2022, were included in this population-based study.

**Results:**

Among the 5153 patients, most were female (74.5%), married (70.8%), and did not hold an academic degree (53.8%). Unemployment (OR: 3.75) and being unmarried (OR: 2.60) were significantly associated with Expanded Disability Status Scale (EDSS)≥6, and the time to progression was shorter in the unemployed group (*P* value: 0.03). There was also a significant negative correlation between the time to progression and the age at disease onset.

**Conclusion:**

The study suggests that providing financial and social support to MS patients and their families through investment could reduce both individual and societal burdens.

## Introduction

 Multiple sclerosis (MS) is a potentially disabling disease affecting different aspects of patients’ life. Its prevalence has been rising in recent years.^1^ In terms of productivity changes, 60 hours could be lost over three months due to MS.^2^ Aside from the probable physical disabilities, the cognitive aspects could also pose a substantial burden to the patients, their close ones, and the society.^3^

 Current evidence suggests a multifactorial model predisposing an individual to MS. Some studies mention culprit genes like HLA-DR15,^4^ besides 110 single nucleotide polymorphisms outside HLA regions,^5^ and female predominance,^6^ while many others highlight the role of environmental factors like Epstein-Barr virus, other viruses like cytomegalovirus and HHV-6, vitamin D, smoking, obesity, or the gene-environment interaction.^7-10^

 Socioeconomic status (SES) is a widely discussed matter in the field of MS. Although the exact definition of “socioeconomic status” is not well established, educational level, employment, income, and insurance coverage are its main components.^11^ It is considered to affect the incidence and progression of the disease,^12-14^ and access to treatment^15^; it is also an aspect of patients’ lives that could be altered due to the disease. However, these associations are not quite straightforward. There are doubts about the exact mechanism and final determinants. Uncertain evidence suggests high SES level as a risk factor for developing MS.^16^ On the other hand, lower SES could be associated with a poorer outcome of the disease.^17^ One study by Mohtasham et al showed that the effect of some SES determinants like income and the education level of the fathers could be related to primary progressive MS (PPMS).^18^

 Iran is a lower-middle-income country as the World Bank reports.^19^ There is evidence of rising MS prevalence and incidence in Iran.^20,21^ The cost of MS was estimated to be $238 124 160 in 2019-2020,^22^ or about US$ 34000 per patient^23^ which seems a notable number for Iran’s level of income. This high economic burden, in addition to international sanctions, could limit the patients’ access to treatments.^24^ To obtain a better understanding of the patients’ status, we investigated the SES determinants of Iranian MS patients. We also studied their relation to disability and disease progression. The results are hoped to shed light on the current condition which could be helpful in future policymaking.

## Materials and Methods

 This population-based study was conducted on all MS patients in the nationwide MS registry of Iran (NMSRI) up to January 8, 2022. This validated system has empowered subsequent policymakers to achieve a superior image of different aspects of disease conditions at a national level through qualified research.^25-27^ MS was diagnosed by neurologists based on McDonald’s criteria 2017.^28^ Registrars registered the data of subjects in 15 cities/ provinces, 12 MS societies, 3 referral hospitals, and 6 private clinics. Included were developed provinces, medium-developed provinces, and undeveloped provinces. The level of development was measured by 25 economic indices and ranked by factor analysis method.^29,30^

 Data on basic characteristics (age, gender, marital status), socioeconomic determinants (family size, education level, employment, owning a home, health insurance), and disease-related variables (MS type (relapsing versus progressive), disease-modifying treatment, Expanded Disability Status Scale (EDSS) (higher or lower than 6), age at the onset of the disease, diagnosis delay since the first symptom, time from the onset of the disease to progression, using assistance equipment (cane, walker, wheelchair), participating in physical rehabilitation programs (physiotherapy, occupational therapy)) were obtained. In Iran, a person who is not employed and is not willing to be employed or is not interested in working in any economic activity is classified as “unemployed”. Typically, homemakers are classified under this category. Therefore, housewife females were entered as unemployed. There is mandatory governmental health insurance for every Iranian individual besides other insurance options with more extensive coverage. Descriptive analysis and logistic regression were performed in IBM^®^ SPSS^®^ version 26. To test normal distribution of quantitative variables, Kolmogorov-Smirnov normality test was adopted. For variables without a normal distribution, Mann-Whitney U test and Spearman rank test were applied as applicable. To build the final regression model, after finding possible statistically significant associations, known risk factors were fixed in the model and a stepwise forward method was used. For first-step univariate regression, *P *value < 0.2; and for the final model, *P *value < 0.05 were considered significant. To assure fulfilling logistic regression assumptions, Box-Tidwell test was used.

 After informing the patients about the goals and structure of the study, they were free to enroll. Different access levels were used to reassure privacy and data security.

## Results

 After excluding incomplete files, data on a total of 5153 MS patients were evaluated. Their characteristics are summarized in Table 1. The majority of cases were female (74.5%), unemployed (65.1%), married (70.8%), and without an academic degree (53.8%).

**Table 1:** Basic Characteristics of the Participants

**Variable**	Number (%)*
Gender (female)	3837 (74.5)
Mean age (SD)	36.3 (9.8)
Employment status	
Employed	1621 (31.5)
Unemployed	3357 (65.1)
Missing	175 (3.4)
Married	3647 (70.8)
Median family size (IQR)	4 (3-4)
Education	
Illiterate	0 (0)
Without academic degree	2770 (53.8)
With academic degree	2300 (44.6)
Missing	83 (1.6)
Housing	
Owning a home	3594 (69.7)
Rental accommodation	1444 (28.0)
Missing	115 (2.2)
No health insurance coverage	79 (1.5)
MS type	
Relapsing	4247 (82.4)
Progressive	830 (16.1)
Missing	76 (1.5)
Mean onset age (SD)	29.3 (8.9)
Median diagnosis delay (IQR), year	≥ 0 (0-1)
Median time to progression (IQR), year	8 (4-12)
Family history of MS	
Positive	926 (18.0)
Negative	4079 (79.2)
Missing	148 (2.9)
EDSS ≥ 6	239 (4.6)
Using assistance equipment	371 (7.2)
Positive history of physical rehabilitation	677 (13.1)

SD, standard deviation; IQR, interquartile range. *For quantitative variables, mean (SD) or median (IQR) are shown.

 Most female patients (n: 2851/3837, 74.3%) were housewives. Among men, 427 (32.4%) were unemployed. Also, 211 patients (4%) were divorced at the time of the study. The mean age of unemployed men (39 ± 12.6) was significantly higher than those with a job (35.8 ± 9.1) (*P *value < 0.001). Out of 88 patients who declared a reason for their divorce, 25 (28%) stated MS as the reason. Having an academic degree was significantly associated with being employed (OR: 3.51, *P *value < 0.001).

 Family history of MS was not significantly associated with any of the demographic, socioeconomic, and disease characteristics.

 Considering EDSS ≥ 6 as the outcome measure, gender and age were fixed in the multivariable model. Of all socioeconomic determinants, unemployment and not being married were significantly associated with EDSS ≥ 6 (Table 2).

**Table 2:** The Output of Final Multivariable Regression Model

**Variable**	**Exp (B) (95% CI)**	***P *****value**
Male gender	2.80 (2.04 – 3.84)	< 0.001
Age (y)	1.11	< 0.001
Not married	2.63 (1.90 – 3.64)	< 0.001
Unemployment	6.15 (3.82 – 9.90)	< 0.001

B, beta coefficient; CI, confidence interval; Exp (B), exponential B that equals to odds ratio (OR) for qualitative. variables.

 Time from the onset of the disease to progression was not normally distributed (*P* value < 0.001). It was found that time to progression was significantly less in the unemployed group (*P* value: 0.03). Besides, a significant negative correlation was found between time to progression and age at disease onset (rho: - 0.22, *P* value < 0.001).

## Discussion

 This study depicts a clear view of the SES of Iranian MS patients.

 The unemployment rate of 65% in general and 32.4% in men seems notable, compared to the reported unemployment rate of 9.4% in Iran in 2020.^31^ The higher mean age of unemployed men (39.3) (compared to 35.8 in men with a job) is another point to consider. Although the difference is not large, it may point to more job losses as time passes. Added to the facts that most Iranian MS cases are in this age range,^32^ many unemployed cases have families of around four, around 54% of the patients do not have an academic degree, and 28% do not own a house, this calls for the attention to the issues of employment and income in this group. This would aid patients in leading an independent life. Besides, solving these problems could reduce the considerable burden of the disease on the society.

 Only a minority (n = 79, 1.5%), did not have any health insurance coverage. As MS treatment and follow-up could be quite expensive in Iran without insurance, the reasons for no coverage in even this small group should be investigated and solved.

 Progression is an important subject in MS, imposing a considerable socioeconomic burden on the patient^33^ and the society.^34^ It is important to delay it, or once started, reduce its speed in order to limit the resulting disabilities. Around 16% of Iranian patients were in the progressive phase. Other studies estimate the frequency of progressive MS at 11% in Kermanshah (western Iran),^35^ and 14% in East Azerbaijan (northeastern Iran).^36^ The prevalence of progressive cases is around 14% in Denmark^37^ and Chile,^38^ around 27% in Germany,^39^ and 8% in Argentina.^40^ The difference in the statistics could be the result of the study population and variable definitions. For example, in the German study, only + 18-year-old cases were included, or in the survey from Argentina, secondary-progressive MS (SPMS), relapsing-remitting MS (RRMS), and clinically isolated syndrome (CIS) cases were considered relapsing-onset versus PPMS cases as progressive-onset MS.

 The observed correlation of the mean onset-age with time to progression is in line with the results of a Swiss study by von Wyl et al,^41^ along with older studies.^42,43^ The higher inflammatory (and less degenerative) nature of the earlier-onset MS is the proposed underlying mechanism.

 About 18% of participants had a family history of MS, which is in line with the results of another study from Iran (19%). However, as predicted for genetic predisposition, regional variances have been found.^44^ We could not find any association between the familial history of MS and the basic characteristics of the patients. However, in a study by Salehi et al, female gender and the RRMS/SPMS phenotypes were more frequent among familial cases but no such association was found regarding EDSS.^44^ A systematic review in 2021 estimated the prevalence of familial MS at around 11%. No association of gender with familial form was found.^45^

 EDSS ≥ 6, indicating significant disability, was significantly higher in males and older patients as predicted.^46^ Its relation to unemployment and not being married is complicated. Both could be considered as possible predisposing factors and also consequent effects of the disease. A French study used European Deprivation Index and showed higher risk of reaching EDSS > 6 in more socioeconomically deprived patients.^47^ In the study by Harding et al, neighborhood-level average income was used as a measure of SES. They showed the higher SES levels could delay disability progression.^12^ From another point of view, job loss could be due to MS-related disabilities which highlights the need for work adaptations.^48^ In our study, unemployed cases progressed sooner than the others. Ware et al, using both neighborhood-level and participant-level indicators (education level), presented evidence that socioeconomic disparity is associated with an enhanced neurodegeneration process.^49^

 With regard to marriage, cultural factors in Iran complicate final decisions based on current findings. Some partnered people may not declare their relationship status. In addition, the reasons for divorce or being single could not be exactly elucidated. Besides, the cultures differ in urban and rural areas and different cities. But generally, it is important to bear in mind that spousal support is crucial for rehabilitation.^49^

 Regarding rehabilitation, around 140 patients with EDSS < 6 use assistance equipment. This could indicate that some cases have to use this equipment in some especial occasions. If proven, this should be taken into consideration in policy making for supporting these patients.

 There is much recent insistence on motor rehabilitation in MS as a therapeutic option that could even change MRI markers.^50^ However, only 13% of our patients used such programs. As there is no precise data on the details of patients’ symptoms in our database, one could comment that not many of the patients might have motor weakness and so benefited from physical therapy or occupational treatment. Another explanation is the relative young age of the patients. One could speculate that as the mean age of the patients rises,^51^ there will be greater need for rehabilitation. Another point regarding MS care and services in Iran is that more than half of the subjects (51.9%) mentioned experiencing high stress level for increased costs of rehabilitation services^24^ while people with MS need long-term rehabilitation care.^52^

 Iran is a country with a heterogeneous socioeconomic structure. In-country (rural to urban areas, small to big cities) immigration, access to medical facilities,^53^ and significant regional inequality^54^ complicate socioeconomic investigations in the country. Besides, this study relies on patients’ reports on variables like employment, housing, marriage history, family history, and using rehabilitation that could be biased. Our study design also bears the common limitations of a database method which may not be the best way to investigate a correlation. It should be emphasized that no final judgement could be made on the causative relationship between presumed determinants and outcome measures in the absence of more robust evidence. In other words, MS could affect and also be affected by socioeconomic performance of the patients. However, in the absence of stronger evidence, this could be the first step. Only including patient-related indicators could be considered another limitation. It is encouraged that neighborhood indicators are investigated in future reports.

 In conclusion, the evidence shows that SES is an important factor in MS disease course. Investments in supporting MS patients and their families, financially and socially, could enhance this group’s physical and mental health, reducing the rate of progression, and decreasing the final burden of the disease on the individuals and the society.
